# The Effects of Dietary Supplements on Asthma and Lung Cancer Risk in Smokers and Non-Smokers: A Review of the Literature

**DOI:** 10.3390/nu11040725

**Published:** 2019-03-28

**Authors:** Naser A. Alsharairi

**Affiliations:** Menzies Health Institute Queensland, Heart, Mind and Body Research Group, Griffith University, 4222 Gold Coast, Australia; naser.alsharairi@gmail.com

**Keywords:** lung cancer, asthma, dietary supplements, current smokers, former smokers, non-smokers

## Abstract

Smoking is one of the major global causes of death. Cigarette smoke and secondhand (passive) smoke have been causally related to asthma and lung cancer. Asthma is a potential risk factor for developing lung cancer in both smokers and non-smokers. Prospective studies and randomized control trials (RCTs) of dietary supplements and lung cancer risk in adult smokers and non-smokers have yielded inconsistent results. A few prospective studies have shown that long-term use of high doses of some supplements, such as retinol, β-carotene, B vitamins, and vitamin E, increase lung cancer risk in current and former smokers. Limited evidence from RCTs suggests that vitamin D supplementation is effective in improving lung function and reducing asthma risk in current/former smokers. The relationship between dietary supplements and lung cancer risk has never before been examined in asthmatic smokers and non-smokers. This short review aims to examine the evidence from existing studies for the effects of dietary supplements on asthma/lung cancer risk and mortality in smokers and non-smokers.

## 1. Introduction

Smoking harms several organ systems of the body and is considered a major risk factor for many diseases (e.g., lung and other cancers, coronary heart diseases and stroke, chronic obstructive pulmonary diseases) that are the largest causes of death among adults worldwide [1,2,3]. Tobacco smoke contains thousands of toxic chemicals, of which 60 are identified carcinogens [4]. Smoking caused nearly 12% of global deaths in 2015, with 52% of those deaths taking place in Russia, India, China, and the USA [3]. It is well known that exposure to second-hand smoke (SHS) is associated with deaths from serious diseases (e.g., ischemic heart disease, chronic obstructive pulmonary disease, stroke, lung cancer) in adults [5,6]. Worldwide, more than 600,000 premature deaths were estimated to have been caused by SHS exposure in 2004. Of these, the rate of female non-smokers (47%) was higher than that of children (28%) and men (26%) [7].

Lung cancer is the first ranked among cancers, accounting for 18.4% of global cancer deaths in 2018. By region, the incidence rates were higher in men than in women, with the highest rates in Micronesia/Polynesia (52.2%), Eastern Europe (49.3%), East Asia (47.2%), and Western and Southern Europe (43%) [8].Cigarette smoking is the main risk factor for lung cancer among men and women worldwide [9], with over 76% (aged ≥50 years) and 49% (aged 15–49 years) of lung cancer deaths in men and 37–42% (aged ≥50 years) of lung cancer deaths in women caused by tobacco use in 2017 [10]. There are several longitudinal and case-control studies that clearly link current and ex-smoking of cigarettes with lung cancer [11,12]. The risk of lung cancer was also caused by other forms of tobacco use, in particular, cigars and pipes/waterpipes [13,14,15]. SHS is recognized as a cause of lung cancer. Several case-control and prospective cohort studies have shown that SHS or passive smoking raises the chance of getting lung cancer in non-smokers, particularly in women [12,16].

Asthma is one of the most prevalent chronic diseases in young adults [17]. Globally, the prevalence of current asthma among adults (aged 18–45 years) varies widely, with the highest prevalence seen in Australia [18]. The global burden of disease (GBD) study reported that 400,000 deaths were caused by asthma in 2015, and the rates were higher in males than in females [19]. Cigarette smoke and SHS exposure are the major triggers of asthma exacerbations in adults. Findings from systematic reviews and meta-analysis of prospective and cross-sectional/case-control studies have confirmed that the risk of asthma has increased in adult current and former smokers [12,20]. Exposure to passive smoke also increased the risk of asthma in adult non-smokers [12]. Asthma has been found to be a risk factor for lung cancer. A few systematic reviews and meta-analyses of prospective and case-control studies have shown that asthma is associated with risk of lung small-cell and squamous cell carcinoma, but not with lung adenocarcinoma. Furthermore, the risk of lung cancer was high in asthmatic smokers and non-smokers [21,22,23].

Dietary supplements are commonly used by cancer patients as they hope these supplements might help to fight their cancer. However, it has been proven that these supplements have the potential to be harmful [24]. A number of prospective and RCT studies that examined the effect of dietary supplements on lung cancer risk and mortality in adult smokers/non-smokers have reported inconsistent results. In a few long-term prospective cohort studies, the risk of lung cancer increased with high doses of retinol supplements in former smokers [25], and with B vitamins (B6, B12) and vitamin E supplements in current smokers [26,27]. Only β-carotene supplements increased the risk of lung cancer among male current smokers in a trial of α-tocopherol and β-carotene supplements [28], but none of the supplements had an effect on post-trial lung cancer risk [29]. In a large trial of retinyl palmitate and β-carotene supplements with a post-trial follow-up of lung cancer, the risk remained increased in former smokers, but overall mortality decreased in current and former smokers [30]. In a large cohort study, multivitamins and other dietary supplements (e.g., ginseng/Q10, herbs/plants, single minerals) taken daily or occasionally had a significant effect in reducing mortality from lung cancer in current smokers [31]. In one cohort study, the risk of lung cancer was increased with vitamin E supplements in non-smokers exposed to side-stream smoke at home/work [32], whereas another study detected no significant effect from vitamin D and calcium supplements [33]. Although multivitamins/other supplements are widely used to prevent chronic diseases [34], the efficacy of these supplements in reducing or treating asthma in adult smokers is unclear. In adult current/former smokers, in whom the use of dietary supplements in the treatment of asthma remains the least studied, vitamin D supplementation tends to improve lung function and decrease risk of asthma [35]. To our knowledge, the risk of lung cancer associated with dietary supplements has never before been studied in asthmatic smokers and non-smokers. Given the significant link between asthma and lung cancer, this link could have health implications for smokers and non-smokers in terms of using dietary supplements. This short review aims to examine existing evidence of the effect of dietary supplements on asthma and lung cancer risk and overall mortality in smokers and non-smokers.

## 2. Method

The PubMed/MEDLINE database was searched for relevant articles up to January 2019, using the following key words: “Lung cancer” AND “supplements” OR “multivitamins” AND “smokers” OR “non-smokers” AND “asthma”. Studies were included if they focused on the effects of supplements and/or vitamin/mineral intake (from both diet and supplements) on asthma and lung cancer risk and total mortality rate, with the overall results stratified by smoking status or groups. Studies with a primary focus on the effects of dietary intake on risk of lung cancer or asthma were not considered. Studies focusing on other allergic/atopic conditions (atopic dermatitis/eczema, allergic rhinitis, hay fever, food allergy) were also not considered, as the extent of the association between these conditions and lung cancer are still questionable, due to inconsistent findings across studies [36,37,38,39,40].

## 3. Results

Nine hundred and forty studies matching these key words were identified. Twenty studies were selected for full review. Details of the selected studies are provided in the following sections and Table 1.

### 3.1. Smoking and Risk of Asthma

The Vitamin D Assessment (ViDA) study was a randomized, double-blind, placebo-controlled trial, designed to examine the effect of vitamin D supplementation on lung function in adults. The assessment used self-administered questionnaires to collect data on demographic variables, use of vitamin D supplements, history and medications for asthma, sun exposure, and smoking. Lung function (FEV1) was measured using a KoKo Trek spirometer. The effect in the vitamin D supplemented group on lung function was greater and significant compared to the placebo group. In particular, a significant improvement in lung function was observed among ever-smokers (current/former), ever-smokers with vitamin D deficiency, and ever-smokers with asthma/chronic obstructive pulmonary disease (COPD). A more significant lung function improvement was also observed among ever-smokers with asthma/COPD than among non-smokers [35].

### 3.2. Smoking and Risk of Lung Cancer

#### 3.2.1. Prospective Cohort Studies

##### Smokers

Three prospective cohort studies performed in the US have used the VITamins and Lifestyle (VITAL) study data. The first study examined associations of long-term, high-dose supplementation (lutein, retinol, vitamin A, β-carotene, and lycopene) with lung cancer risk among men and women. The design involved asking the participants to complete a baseline questionnaire, which sought information about multivitamin/supplement use during the last 10 years. Results showed that use of retinol supplementation for ≥4 years was associated with a significant 53% increased risk of total lung cancer and 80% increased risk of non-small cell cancer. Using β-carotene supplementation for ≥4 years was associated with an increased risk of small cell lung cancer. Ten-year average daily intakes of supplemental retinol in high doses (>1200 mcg/day) were associated with an elevated lung cancer risk of 46% among former smokers (quit ≥10 years) [25]. The second study which examined associations between long-term, high-dose supplementation of multivitamins/other supplements and risk of lung cancer, showed that current smokers with a 10-year use of high dose vitamin E supplements (>215 mg/day) had a 59% increased risk [26]. The third study, which examined associations between long-term, high-dose supplementation of B vitamins and lung cancer risk among current smokers, showed that 10-year use of supplemental B6 (>20 mg/day) and B12 (>55 µg/day) in high doses was associated with an increased risk of lung cancer [27].

A large national cohort study of cancer patients examined whether cod liver oil and other dietary supplements used before diagnosis improved patients’ survival. Among lung cancer patients prior to their diagnosis, current smokers achieved higher than non- or former smokers. Validated semi-quantitative questionnaires including Food Frequency Questionnaire (FFQ) were used to assess demographic variables and food intake/supplements over the past 12 months. Patients were classified as supplement occasional users, whole year daily users, seasonal daily users, and non-users. Results found that cod liver oil and other dietary supplements improved lung cancer survival. Whole-year daily use of cod liver oil was associated with a 44% decrease in death. Daily and occasional users of other dietary supplements were associated with a 30%and 45% decrease in death, respectively [31]. A large prospective cohort study examined the association of mineral intake from diet/supplements with lung cancer risk, and showed that among current smokers, a high intake of Ca from diet (>1362 mg/day) was associated with a 14% reduction in risk, whereas a high intake of Mg from diet (>449 mg/day) was associated with a 28% increase in risk. Mineral intake from supplements had no effect on lung cancer risk [41]. A study examining associations between multivitamin/mineral use and the risk and mortality from several cancers among men and women reported no association of multivitamin/mineral use with risk and mortality of lung cancer among current or former smokers [42].

##### Non-Smokers

Two prospective cohort studies carried out on Chinese female non-smokers used the Shanghai Women’s Health Study data. The first study examined associations between α-tocopherol (vitamin E) intake from diet/supplements and lung cancer risk. Validated self-administered FFQs were used at baseline and follow-up (every 2–3 years) to measure the frequency of eating food during the month prior or taking vitamin/mineral supplements (multivitamins, calcium, vitamin C, B, E, and A) for more than two months. Women were classified as never exposed or exposed to side-stream smoke at home/work. During the follow-up period, 481 lung cancer cases were identified. Results showed that women who were exposed to side-stream smoke at home/work were twice as likely to develop lung cancer when using vitamin E supplements [32]. In another study examining associations between calcium/vitamin D intake from diet/supplements and lung cancer risk among women exposed to passive smoke at home/work, no association between the use of calcium or vitamin D supplements and risk of lung cancer was observed [33].

#### 3.2.2. Randomized Controlled Trials/Double-Blind, Randomized, Placebo Controlled Trials

##### Smokers

A double-blind, randomized, placebo-controlled trial carried out in 40 centers examined the associations between supplemental vitamin D plus calcium (CAD supplementation) and lung cancer risk, and whether the ratio of baseline Ca:Mg intake modified such associations. Results revealed no significant associations between supplemental CAD and lung cancer risk in both groups. However, the risk of lung cancer decreased by 35% among current smokers with CAD supplementation and a low Ca:Mg intake ratio (≤2.53), but not among those with a high Ca:Mg intake ratio (>2.53) [43]. The goal of the randomized double-blind, placebo-controlled trial, the Nutritional Prevention of Cancer Trial, was to evaluate the effectiveness of selenium supplementation in reducing the incidence of cancer among patients diagnosed with prostate, colorectal, skin, breast, head and neck, lymphoma, and lung cancer. The trial was successful in significantly reducing risk of prostate cancer. The trial also documented a non-significant 26% reduction in lung cancer risk with selenium supplementation among current and former smokers [44].

A systematic review and meta-analysis of four double-blind, randomized, placebo-controlled trials reported a significant 24% increase in lung cancer risk in current smokers receiving a high-dose of β-carotene from supplements [45]. The Alpha-Tocopherol, Beta-Carotene Cancer Prevention (ATBC) Study was a double-blind, randomized, placebo-controlled trial that examined the effect of α-tocopherol and β-carotene supplementation on lung cancer risk. Results revealed no effect of 50 mg/day α-tocopherol (vitamin E) supplement on the risk of lung cancer, but a 25% increased risk associated with 20 mg/day β-carotene supplement was observed among male current smokers (≥20 cigarettes/day) [28]. In the 18 years of post-intervention follow-up for the same study, 2881 cases were diagnosed with lung cancer, and 16% of all deaths were due to lung cancer. Neither α-tocopherol nor β-carotene supplements had an effect on post-intervention lung cancer risk and mortality [29]. A recent study using the ATBC data assessed whether cigarette tar and nicotine levels modified the association between β-carotene supplementation and lung cancer risk. Smokers were classified as ultralight (<7 mg tar), light (8–14 mg tar), regular (15–20 mg tar), non-filtered (>20 mg tar), ventilated filter (≤0.8 µg nicotine), unventilated filter (>0.8 to ≤ 1.3 µg nicotine), and no filter (>1.3 µg nicotine). Results found a significant association between β-carotene supplementation and lung cancer risk, regardless of the nicotine or tar levels. The lung cancer risk increased by 31%, 18%, 15%, and 22% (tar levels), and by 23%, 15%, and 22% (nicotine levels) among men when supplemented with β-carotene [46]. The EUROSCAN study, a randomized intervention trial, examined the effect of supplementation of *N*-acetylcysteine (an antioxidant in supplement form) and retinyl palmitate (vitamin A) on lung, neck, and head cancer survival among cancer patients who were mainly current or former smokers. Results showed no effect on lung cancer risk after two years’ use of *N*-acetylcysteine and/or retinyl palmitate supplements [47].

The Women’s Health Initiative (WHI) was a prospective observational and randomized placebo-controlled trial (1993–2010), aimed at examining the association between vitamin D intake as well as calcium/vitamin D supplementation and lung cancer risk, and whether vitamin A intake modified such associations among postmenopausal women. Vitamin D intake was assessed using validated self-administered FFQs. Results showed that the risk of lung cancer was reduced by 13%, although not statistically significant, among women exposed to the calcium/vitamin D intervention, compared to those in the placebo or not exposed to trial. The risk of lung cancer was higher in current smokers with calcium/vitamin D supplementation and a high total vitamin A intake (≥3000 µg/day retinol activity equivalents (RAE)) than with a low total vitamin A intake (<3000 µg/day RAE) [48]. A case-cohort study within the Carotene and Retinol Efficacy Trial (CARET, 1988–2005) was designed to examine the association of estimated vitamin D from food or dietary supplements with lung cancer risk, and whether CARET active intervention (25,000 IU retinyl palmitate plus 30 mg β-carotene/day vs. no intervention) and total vitamin A intake (≥1500 µg/day RAE vs. <1500 µg/day RAE) modified such associations among current and former smokers. A self-administrated FFQ was used to assess total vitamin D and vitamin A intake, whereas an inventory method during clinical visits was used to assess dietary supplements. The study showed a non-significant association between a high intake of vitamin D (≥800 IU/day), a 74% reduction in lung cancer risk among former smokers, and 7% reduction among current smokers. Furthermore, a non-significant 75% reduction in risk was observed among former smokers who had a high intake of vitamin D (≥400 IU/day) and received the CARET active intervention. However, a significant 62% reduction in risk was observed among current smokers who had a high intake of vitamin D (≥400 IU/day) and vitamin A (≥1500 µg/day RAE) [49]. Goodman et al. [30] in the CARET study assessed whether six post-trial years of retinyl palmitate and β-carotene supplementation could reduce lung cancer incidence and total mortality rates after patients stopped taking the supplements, due to an increase in incidence and mortality rate. Intervention patients in the post-trial phase were reassigned to receive a daily dose of 25,000 IU retinyl palmitate and 30 mg β-carotene/day. During the post-trial phase, current smokers had a 20% lower lung cancer risk and a 39% lower mortality rate than those in the trial phase. Compared to former smokers in the trial phase, those in the post-trial phase had a 31% increased risk but a 15% reduced mortality rate.

#### 3.2.3. Case-Control Studies

##### Non-Smokers

A study using the Women’s Health Initiative (WHI) data aimed to examine whether the associations between 25(OH)D concentration and lung cancer risk differed in a calcium/vitamin D trial intervention (intervention group vs. placebo group/non-participants in the trial) among never-smoking postmenopausal women. Two case-control pairs were selected (lung cancer cases vs. control non-smokers). In the group of never-smoking women, an effect for the calcium/vitamin D supplementation (400 IU vitamin D_3_ plus 1 g Ca/day) on such associations was observed. Women with high serum 25(OH)D concentrations (≥50 nmol/L) who received the trial supplement were associated with a statistically non-significant 58% reduced lung cancer risk [50].

## 4. Conclusions

This review found contradictory results across studies examining the effects of dietary supplements on lung cancer risk and mortality in adult smokers/non-smokers. This makes it difficult to draw firm conclusions. In adult smokers, long-term use of high doses of retinol, β-carotene, B vitamins, and vitamin E supplements do not appear to have any protective effects against lung cancer risk among current/former smokers in prospective studies. Supplementation with β-carotene appeared to increase lung cancer risk and mortality in the trial phase among male current smokers, but β-carotene and α-tocopherol supplements had no effect in the post-trial phase. β-carotene and retinyl palmitate supplements for several years post-trial resulted in increased lung cancer risk among former smokers, but decreased mortality among current and former smokers. Taking multivitamins and other supplements (ginseng/Q10, herbs/plants) daily or occasionally has been linked to decreased lung cancer mortality among current smokers. Vitamin D/calcium supplementation together with high vitamin A and calcium/magnesium intake resulted in significant increases in lung cancer risk among current smokers. Mineral supplements/multivitamins, *N*-acetylcysteine and retinyl palmitate had no effect on lung cancer risk among current/former smokers. In female non-smokers, vitamin E supplementation had been linked to increased lung cancer risk, but vitamin D/calcium supplements had no effect on risk. Overall, supplementation with ginseng/Q10, herbs/plants, and β-carotene/retinyl palmitate may decrease the risk of dying from lung cancer. Vitamin E supplementation does not provide any protection among patients with lung cancer who never smoked. The high intake of vitamin A or calcium/magnesium together with vitamin D/calcium supplements is not advocated. Lack of any effect of supplements with multivitamins, *N*-acetylcysteine, retinyl palmitate, and vitamin D/calcium in prospective studies makes it difficult to recommend that these supplements would be helpful in protecting against lung cancer. This review suggests caution in recommending long-term, high-dose supplements that contain β-carotene, retinyl palmitate, vitamin E, B, vitamins (B6, B12) for patients with lung cancer, especially current and former smokers. To conclude, it is important to evaluate safety and effectiveness of dietary supplement use by adult smokers and non-smokers before, during, and after lung cancer treatment.

This review found limited evidence for the effects of vitamin D supplements on asthma risk in adult smokers. Only one RCT found vitamin D supplementation did significantly improve lung function and reduce asthma risk in current/former smokers. Further investigations will be needed to examine the effects of other supplements on asthma risk/mortality in adult smokers/non-smokers. Clearly, there is still a gap in knowledge with respect to the effects of dietary supplements on lung cancer risk/mortality in asthmatic smokers and non-smokers. Future trials and prospective studies of these supplements are needed to detect their benefits and harms.

## Figures and Tables

**Table 1:** Characteristics of studies included in the review.

Reference	Country	Subject Characteristics	Study Design	Outcome	Supplement Intake	Study Duration/Follow-Up	Main Reported Results
Sluyter et al. [35]	New Zealand	Total subjects = 442 (vitamin D group = 226; placebo = 216) Never-smokers (vitamin D group = 122; placebo = 103) Ever-smokers (current/former) (vitamin D group = 104; placebo = 113) Ever-smokers+ vitamin D deficient (vitamin D group = 26; placebo = 28) Ever-smokers+ asthma/COPD (vitamin D group = 25; placebo = 35) Age = 50–84 years	RDBPC	Lung function, asthma/COPD	Vitamin D3 capsules 200,000 IU at baseline, followed by monthly 100,000 IU or monthly placebo soft-gel oral capsules	1 year	Across ever-smoker subgroups, the effect of vitamin D supplementation compared to placebo was significant. Ever-smokers (β = 57 mL, *P* = 0.03), ever-smokers with vitamin D deficiency (β = 122 mL, *P* = 0.04), ever-smokers with asthma/COPD (β = 160 mL, *P* = 0.004). A significant improvement in lung function was observed among ever-smokers with asthma/COPD over non-smokers (*P* = 0.0005).
Satia et al. [25]	US	Total subjects = 77,126 Lung cancer cases = 521 (75% were non-small lung cancer), non-smokers = 42, former smokers (quit ≥10 years) = 226, former smokers (quit <10 years) = 93, current smokers = 155 Non-cancer controls = 76,605 Age = 50–76 years	Prospective cohort study	LC risk	Lutein, retinol, vitamin A, β-carotene, and lycopene	10 years	Use of retinol supplement was associated with a statistically significant increased risk for former smokers (quit ≥ 10 years) (HR = 1.46, 95% CI = 0.93 to 2.29). None of the other supplements were associated with cancer risk in current/former smokers
Slatore et al. [26]	US	Total subjects = 77,126 Lung cancer cases = 521, non-smokers = 42, former smokers (quit ≥10 years) = 226, former smokers (quit <10 years) = 93, current smokers = 155 Non-cancer controls = 76,605 Age = 50–76 years	Prospective cohort study	LC risk	Multivitamins, folate, vitamin C, and E	10 years	Use of vitamin E supplement was associated with a statistically significant increased risk for current smokers (HR = 1.59, 99% CI = 1.50 to 2.41). All other supplements were not associated with a reduced or increased risk in current/former smokers.
Brasky et al. [27]	US	Total subjects = 77,118 Lung cancer cases = 808, non-smokers = 60, former smokers (quit >10 years) = 334, former smokers (quit <10 years) = 152, current smokers = 251 Non-cancer controls = 76,310 Age = 50–76 years	Prospective cohort study	LC risk	Vitamin B6, B12, and B9/folic acid	10 years	Use of supplemental B6 (HR = 2.93, 95% CI = 1.50 to 5.72) and B12 (HR = 3.71, 95% CI = 1.77 to 7.74) was associated with LC risk among current smokers only. In contrast, use of vitamin B9 was not associated with LC risk among current/former smokers.
Wu et al. [32]	China	Total subjects = 72,829 female non-smokers Age = 40–70 years	Prospective cohort study	LC risk	Vitamin E	12 years	Vitamin E supplement was associated with LC risk among females likely exposed to side-stream smoke (HR = 2.06, 95% CI = 1.31 to 3.23).
Takata et al. [33]	China	Total subjects = 71,267 female non-smokers Age = 40–70 years	Prospective cohort study	LC risk	Calcium/vitamin D	12 years	No association was observed between calcium/vitamin D supplement and LC risk among female who had passive smoking exposure at home/work.
Skeie et al. [31]	Norway	Total subjects = 2997 female cancer patients with solid tumors Lung cancer patients = 217 Non-smokers = 3.9% Former smokers = 12.6% Current smokers = 83.5% Age = mean ~58 years	Prospective cohort study	LC mortality	Multivitamins, ginseng/Q10, herbs/plants, minerals, vitamin B, C, and E	3 years	Whole year daily use of cod liver oil (RR = 0.56, 95% CI = 0.35 to 0.92) and daily (RR = 0.70, 95% CI = 0.49 to 0.99) and occasional (RR = 0.55, 95% CI = 0.31 to 0.97) users of other dietary supplements were associated with a statistically significant decreased death.
Mahabir et al. [41]	US	Total subjects = 482,875 men and women Non-smokers = 170,401 Former smokers = 237,216 Current smokers = 57,142 Age = 50–71 years	Prospective cohort study	LC risk	Calcium, magnesium, zinc, iron, selenium, copper	7 years	Mineral supplements were not associated with risk in current/former smokers.
Park et al. [42]	US	Total subjects = 182,099 men and women Former smokers (supplement users—men) = 51.9% Former smokers (supplement users—women) = 29.5% current smokers (supplement users—men) = 16.5% Current smokers (supplement users—women) = 13.2% Age = 45–75 years	Prospective cohort study	LC risk and mortality	Multivitamins/mineral	11 years	Multivitamin/mineral supplements were not associated with mortality and LC risk in current/former smokers.
Tao et al. [43]	US	Total subjects = 36,382 postmenopausal women(CaD group = 18,176; placebo = 18,106) Non-smokers (CaD group = 9325; placebo = 9428) Former smokers (CaD group = 7255; placebo = 7133) Current smokers (CaD group = 1405; placebo = 1356) Age = 50–79 years	RDBPC	LC risk	Daily dose of vitamin D (400 IU D3) plus calcium (1000 mg calcium carbonate) or daily placebo multivitamin tablets	11 years	After the follow-up period, the CaD supplementation was not associated with LC risk among current/former smokers. However, the CaD supplementation increases the risk among current smokers with a high Ca:Mg ratio (> 2.53) (HR = 1.36, 95% CI = 0.78 to 2.36).
Duffield-Lillico et al. [44]	US	Total subjects = 1312 cancer patients Lung cancer patients = 60 (selenium group = 25; placebo = 35) Non-smokers (selenium group = 34; placebo = 30) Former smokers (selenium group = 39; placebo = 40) Current smokers (selenium group = 27; placebo = 30) Age = mean ~61 years	RDBPC	LC risk	Daily dose of 200 µg selenium or a placebo	13 years	Selenium supplementation reduced LC risk among current/former, although the reduction was not statistically significant (RR = 0.74, 95% CI = 0.44 to 1.24).
Tanvetyanon and Bepler [45]	US and Finland	Total subjects = 109,394 lung cancer patients Former smokers = 15,076 Current smokers = 24,109	Systematic review/meta-analysis of four RDBPC	LC risk	Daily dose of 20–30 mg β-carotene or a placebo	2–12 years across studies	β-carotene supplementation increased LC risk among current smokers but not former smokers (OR = 1.24, 95% CI = 1.10 to 1.39).
Albanes et al. [28]	Finland	Total subjects = 29,133 male current smokers Age = 50–69 years	RDBPC	LC risk	α-tocopherol acetate (50 mg/day), β-carotene (20 mg/day), both β-carotene and α-tocopherol, or placebo	5–8 years	β-carotene supplementation increased LC risk (RR = 1.25, 95% CI = 1.07 to 1.46). However, α-tocopherol had no effect.
Virtamo et al. [29]	Finland	Total subjects = 25,563 male current smokers (≥18 cigarettes/day) Age = 50–69 years	RDBPC	LC risk/mortality	α-tocopherol acetate (50 mg/day), β-carotene (20 mg/day), both β-carotene and α-tocopherol, or placebo	18 years	β-carotene and α-tocopherol supplementation had no effect on LC risk/mortality.
Middha et al. [46]	Finland	Total subjects = 29,133 male current smokers β-carotene supplementation group: Ultra-light cigarettes = 1359, light = 2224, regular = 9565,nonfiltered = 1421 Ventilated filtered = 3639, unventilated filtered = 9509, no filter = 1412 Age = 50–69 years	RDBPC	LC risk	β-carotene (20 mg/day) or placebo	5–8 years	β-carotene supplementation increased LC risk among male current smokers, regardless of tar/nicotine content of cigarettes smoked. Ultra-light = (HR = 1.31, 95% CI = 1.91 to 1.89) Light = (HR = 1.18, 95% CI = 0.89 to 1.57) Regular = (HR = 1.15, 95% CI = 1.01 to 1.31) Non-filtered = (HR = 1.22, 95% CI = 0.91 to 1.64) Ventilated filtered = (HR = 1.23, 95% CI = 0.98 to 1.54) Unventilated filtered = (HR = 1.15, 95% CI = 1.01 to 1.31) No filter = (HR = 1.22, 95% CI = 0.91 to 1.64)
Van Zandwijk et al. [47]	Europe (not specified)	Total subjects = 2592 cancer patients Lung cancer patients = 1023 (40% with non-small cell lung cancer) Non-smokers = 168 Current/former = smokers = 2405 Age = mean ~61 years	RCT	LC risk	*N*-acetylcysteine (600 mg/day for two years), retinyl palmitate (300,000 IU/day for the 1^st^ year plus 150,000 IU/day for the 2^nd^ year), both *N*-acetylcysteine and retinyl palmitate or no treatment	2 years	*N*-acetylcysteine and retinyl palmitate supplements were not found to be effective in reducing LC risk among smokers.
Cheng et al. [48]	US	Total subjects = 128,779 postmenopausal women Lung cancer patients = 1771 Age = 50–79 years	RDBPC	LC risk	Daily 400 IU of vitamin D_3_ and 1 g of Ca or placebo	7 years	CaD supplementation reduced LC risk among women, although the reduction was not statistically significant (HR = 0.87, 95% CI = 0.70 to 1.07). The CaD supplementation increases the risk among current smokers with a high total vitamin A intake (≥3000 µg/day RAE) (HR = 2.26, 95% CI = 1.02 to 5.01).
Cheng et al. [50]	US	Total subjects = 596 postmenopausal women 298 lung cancer cases vs. 298 control non-smokers Age = 50–70 years	Case-control study	LC risk	Calcium/vitamin D	1 year	Non-smoker women with high serum 25(OH)D concentrations at baseline and exposure to the CaD trial intervention were associated with a low risk of LC (OR = 0.42, 95% CI = 0.16 to 1.14).
Cheng et al. [49]	US	Total subjects = 1428 men and women 749 lung cancer cases and 679 non-cancer controls Former smokers = 222 Current smokers = 527 Age = 50–69 years	Case-cohort design	LC risk	CARET active intervention: 25,000 IU retinyl palmitate plus 30 mg β-carotene/day	17 years	Former smokers with high vitamin D intake and received the CARET active intervention were associated with a low risk of LC (HR = 0.25, 95% CI = 0.08 to 0.76).
Goodman et al. [30]	US	Total subjects = 13,447 lung cancer patients CARET intervention group = 6902, placebo group = 6545 Former smokers = 6447 Current smokers = 7000 Age = 50–69 years	RCT	LC risk/mortality	25,000 IU retinyl palmitate plus 30 mg β-carotene/day or placebo	6 years	Current smokers had a lower LC risk (1.22 vs. 1.42; RR = 1.22, 95% CI = 0.98 to 1.51) and a lower mortality rate (1.27 vs. 1.66; RR = 1.27, 95% CI = 0.99 to 1.64) than those in the trial phase. Former smokers had an increased risk (1.11 vs. 0.80; RR = 1.11, 95% CI = 0.85 to 1.47) and a lower mortality rate (1.12 vs. 1.27; RR = 1.12, 95% CI = 0.83 to 1.52) than those in the trial phase.

Abbreviation: RDBPC, randomized double blind placebo control; RCT, randomized control trial; LC, lung cancer; HR, hazard ratio; RR, relative risk; OR, odds ratio; CI, confidence interval.
